# Two human metabolites rescue a *C. elegans* model of Alzheimer’s disease via a cytosolic unfolded protein response

**DOI:** 10.1038/s42003-021-02218-7

**Published:** 2021-07-07

**Authors:** Priyanka Joshi, Michele Perni, Ryan Limbocker, Benedetta Mannini, Sam Casford, Sean Chia, Johnny Habchi, Johnathan Labbadia, Christopher M. Dobson, Michele Vendruscolo

**Affiliations:** 1 Yusuf Hamied Department of Chemistry, Centre for Misfolding Diseases, University of Cambridge, Cambridge, UK; 2 Department of Genetics, Evolution and Environment, Institute of Healthy Ageing, University College London, London, UK; 3 Present Address: The California Institute for Quantitative Biosciences (QB3-Berkeley), University of California, Berkeley, CA USA; 4 Present Address: Department of Chemistry and Life Science, United States Military Academy, West Point, NY USA

**Keywords:** Molecular biology, Neuroscience

## Abstract

Age-related changes in cellular metabolism can affect brain homeostasis, creating conditions that are permissive to the onset and progression of neurodegenerative disorders such as Alzheimer’s and Parkinson’s diseases. Although the roles of metabolites have been extensively studied with regard to cellular signaling pathways, their effects on protein aggregation remain relatively unexplored. By computationally analysing the Human Metabolome Database, we identified two endogenous metabolites, carnosine and kynurenic acid, that inhibit the aggregation of the amyloid beta peptide (Aβ) and rescue a *C. elegans* model of Alzheimer’s disease. We found that these metabolites act by triggering a cytosolic unfolded protein response through the transcription factor HSF-1 and downstream chaperones HSP40/J-proteins DNJ-12 and DNJ-19. These results help rationalise previous observations regarding the possible anti-ageing benefits of these metabolites by providing a mechanism for their action. Taken together, our findings provide a link between metabolite homeostasis and protein homeostasis, which could inspire preventative interventions against neurodegenerative disorders.

## Abstract (web summary)

Joshi et al. identify two human metabolites, carnosine and kynurenic acid, that rescue a *C. elegans* model of Alzheimer’s disease by inhibiting the aggregation of the amyloid beta peptide in vivo. They find that these metabolites trigger a cytosolic unfolded protein response through the transcription factor HSF-1 and molecular chaperones DNJ-12 and DNJ-19, thus providing mechanistic links between metabolite homeostasis and protein homeostasis to further insights into interventions against neurodegenerative diseases.

## Introduction

Alzheimer’s disease (AD) is a complex disorder characterized by the presence of aberrant protein deposits in brain tissues^1–3^. Although the molecular origins of this disease are still to be firmly established, it is generally recognized that the presence of protein deposits is associated with the dysregulation of the protein homeostasis network^4,5^. In turn, this system is part of a wider cellular homeostasis system, which includes a variety of other components, including the metabolite homeostasis system^6,7^.

Metabolites affect key steps in cellular pathways where they act as substrates for enzymes or as signaling molecules for the activation of biochemical pathways. For example, caloric restriction and intermittent fasting retard structural and functional decline during aging in laboratory rodents and monkeys^8–10^. Moreover, signaling pathways have evolved to respond to cellular stresses in aging, with the AMPK and mTOR pathways being widely studied in cellular development and ageing^11–14^. It is therefore important to understand how neurodegenerative disorders associated with aging, such as AD, are linked with aberrations in cellular homeostasis. The specific hypothesis that we investigate here is whether the dysregulation of metabolite levels is associated with the aberrant aggregation of proteins in AD. We approach this by testing if supplementing the dysregulated metabolites may prevent or clear aggregation of misfolded proteins.

**Fig. 1:**
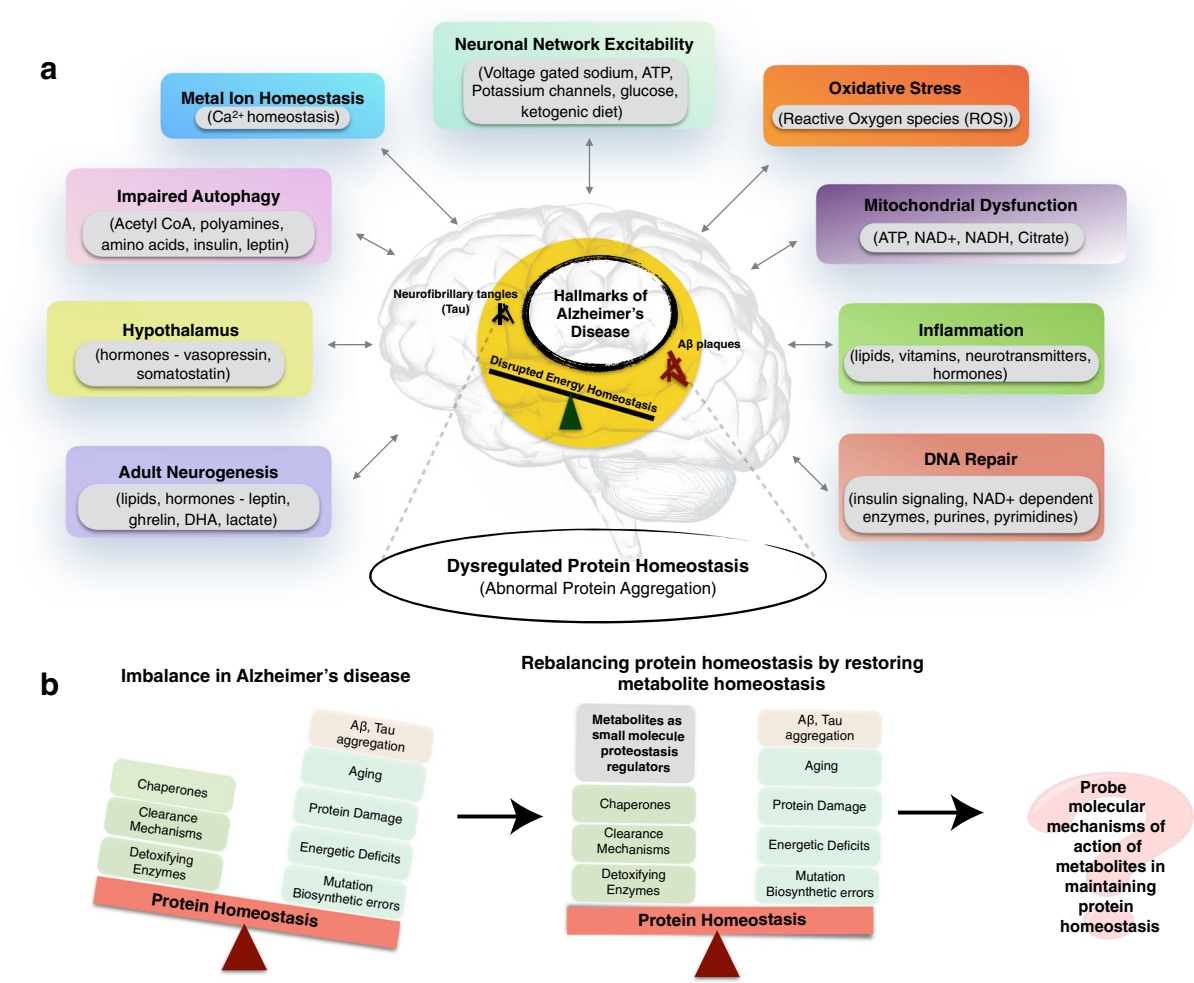
Metabolite homeostasis and protein homeostasis in Alzheimer’s disease (AD). **a** AD is associated with a wide range of dysfunctions in cellular homeostasis (colored boxes). A variety of endogenous metabolites are involved in regulating these cellular processes (gray box insets). **b** Small molecule endogenous metabolites can modulate the protein aggregation processes associated with the disease. By designing systematic studies we can uncover the exact molecular mechanisms by which they balance protein homeostasis.

The impairment of various biological processes contributes to the onset and progression of AD, including DNA repair, inflammation, mitochondrial function, oxidative stress, neuronal network excitability, metal ion homeostasis, autophagy, hypothalamic regulation, adult neurogenesis, and protein homeostasis (Fig. 1)^1,15–28^. Small molecule metabolites play key roles in these biochemical processes, but it is still not known in detail which among these roles are first dysregulated and are associated with the pathophysiological onset and progression of the disease.

In this context, whether the phenomenon of protein misfolding and aggregation is a cause or a consequence of the impairment in these biological processes is yet to be firmly established. A wide range of studies has indicated that protein aggregation can directly result in activation of the downstream biochemical cascades that significantly advance the pathology of AD^29^. The toxic aggregate species are recognized to be Aβ and Tau oligomers, whose abnormal interaction with cellular components can impair neuronal functions^29,30^. Given our present understanding of AD, slowing or inhibiting protein aggregation may contribute to preventing the advancement of the disease. Thus, understanding which pathways contribute towards this objective can offer insightful windows of opportunity for preventative and therapeutic interventions, as well as provide guidelines for sustainable ways of living (e.g. lifestyle factors, exercise, and diet) that suppress AD and promote healthy aging.

In healthy organisms, protein aggregation is prevented by specific branches of the protein homeostasis system, in particular the cytosolic heat shock response (HSR), for cytoplasmic folding and misfolding, and the unfolded protein responses (UPR) in the endoplasmic reticulum and mitochondria^31,32^. Many heat shock proteins from the HSR are molecular chaperones that guide the conformation of proteins during biogenesis and prevent the misfolding and aggregation that interfere with cellular function^21^. Since the cytoplasm is also a pool of various metabolites produced and shuttled across biochemical networks, it is expected that their levels and threshold concentrations in the cell may have an effect as small molecule regulators on the protein homeostasis network, thus keeping aggregation-prone proteins in their soluble state. Patel and colleagues showed that ATP enhances  protein solubility at physiological concentrations (mM range)^33^. One can thus envisage that other endogenous metabolites may also contribute to the modulation of protein aggregation.

Enhanced expression of molecular chaperones, which is primarily regulated by the transcription factor heat shock factor 1 (HSF-1), has been shown to restore protein homeostasis in a variety of protein misfolding disease models, suggesting that this mechanism may represent a promising target for preventative and therapeutic approaches^34^. Calamini et al. identified new classes of small-molecule protein homeostasis regulators that induce HSF1-dependent chaperone expression and restore protein folding in multiple protein conformational disease models. These beneficial effects on proteome stability are mediated by HSF1, FOXO, NRF2, and the chaperone machinery^34^. In fact, endogenous metabolites are also small molecules that regulate key biological processes. Since the levels of these metabolites can change in response to environmental cues, it is important to understand their direct effects on protein homeostasis.

In this work, we asked whether or not endogenous metabolites can influence the aggregation of proteins, and if they do, what is their mechanism of action. Is it a direct influence by binding to the monomeric, oligomeric and fibrillar species or aggregates, or is it indirect by acting on various other cellular homeostatic mechanisms? Through our investigations, we identified two human endogenous metabolites that trigger a cytoplasmic unfolded protein response by increasing the levels of HSF-1, and downstream HSP40 co-chaperones J-proteins, ultimately resulting in the clearance of Aβ aggregates in a *C. elegans* model of AD.

## Results

### Identification of endogenous metabolites as small molecule protein homeostasis regulators

**Fig. 2:**
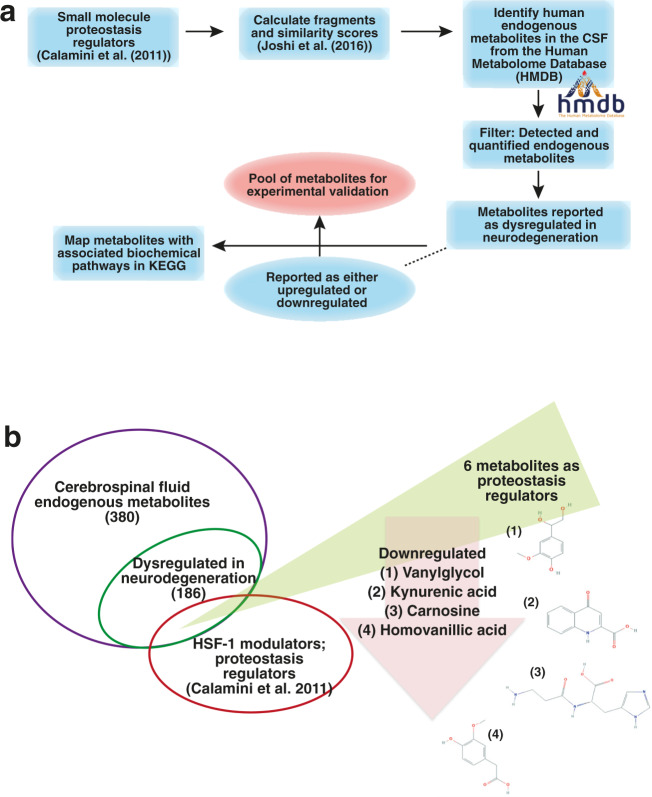
Identification of endogenous metabolites as protein homeostasis regulators that are dysregulated in AD. **a** Schematic of the computational fragment-based strategy that we used to identify endogenous metabolites from the Human Metabolome Database (HMDB) as candidate protein homeostasis regulators that are also dysregulated in AD. **b** Using this approach, we identified six candidate metabolites that serve as protein homeostasis regulators (Table 1, Supplementary Fig. 1 and Supplementary Data 1).

In order to identify metabolites associated with AD, we started by calculating fragments and similarity scores^35^ (see the “Methods“ section) of the small molecules reported as protein homeostasis regulators by Calamini et al.^34^ (Fig. 2a). Using these fragments, we screened the Human Metabolome Database (HMDB)^36^ for metabolites detected in the cerebrospinal fluid (CSF). As it is in direct contact with the extracellular space of the brain, the CSF provides a window on brain metabolism by reflecting the biochemistry of the brain^37^ in the form of its associated metabolites. In this work, we only considered endogenous metabolites, as we wanted to look into molecules active in primary cellular mechanisms, not involving exogenous metabolic products. Our reasoning is that focussing on endogenous metabolites can help identify fundamental cellular processes that are compromised in neurodegeneration as a response to the environment and/or cellular insults.

Since the detection and quantification of metabolites in body fluids is challenging, we filtered only those metabolites that were extensively detected and quantified. We then used the KEGG database^38^ to identify biochemical pathways associated with these metabolites, and further, the metabolites that are particularly dysregulated in neurodegeneration (Supplementary Data 1). In the case of dysregulation, the levels of upregulation and downregulation of these metabolites are available from the HMDB and associated literature available therein. We then performed a literature search to corroborate the dysregulated status of the metabolites in neurodegeneration. We ranked this status as an association score, by performing a PubMed search (see the “Methods” section) (Supplementary Data 1). In principle, the association score describes the association of AD with the identified endogenous metabolite across studies reported in PubMed. Classifying the identified metabolites into upregulated and downregulated gives an insight into the effects of their normal concentrations on maintaining cellular homeostasis and their dysregulation in disease.

We found 186 of the 380 endogenous CSF metabolites to be dysregulated in neurodegeneration (Supplementary Data 1). We then focused on six of these metabolites: 1-methylhistidine, homovanillic acid, melatonin, l-carnosine, vanylglycol, and kynurenic acid (Table 1), which were selected because they shared overlapping fragments with known HSF-1 protein homeostasis regulators (Fig. 2b). We then investigated experimentally their possible roles in modulating the aggregation of Aβ42, the highly cytotoxic 42-residue form of Aβ.

**Table 1:** Dysregulated levels of six endogenous metabolites that we identified as protein homeostasis regulators in this study, as reported in the literature (PubMed ID) and HMDB.

Endogenous metabolite	Regulation	Human Metabolome Database Reference/PubMed ID
1-methylhistidine	Upregulated, Downregulated	HMDB0000001
Homovanillic acid	Downregulated	HMDB0000118
Melatonin	Downregulated	HMDB0001389; 15725334, 14671188, 9920102, 12887656
Vanylglycol	Downregulated	HMDB0001490; 9880039
l-carnosine	Downregulated	HMDB0000033, HMDB0000745; 17031479, 17522447
Kynurenic acid	Upregulated, Downregulated	HMDB0000715; 31376341, 32276479, 17023091, 24855376

### Carnosine and kynurenic acid rescue a *C. elegans* model of AD

**Fig. 3:**
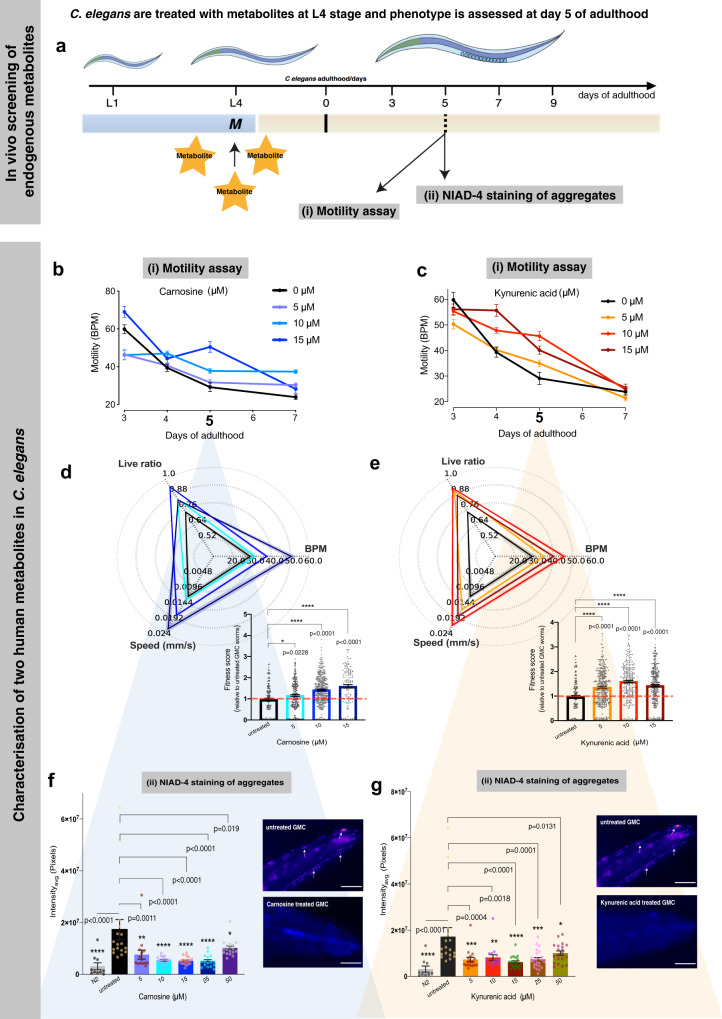
Carnosine and kynurenic acid prevent Aβ42 toxicity in a *C. elegans* model of AD (GMC). In vivo screening strategy to identify endogenous metabolites that inhibit Aβ42 aggregation is illustrated in (**a**). We screened six identified candidate metabolites in a *C. elegans* model of Alzheimer’s disease. Metabolites (depicted as M) were fed at the L4 stage of GMC worms and their effects were assessed at day 5 of adulthood through a (i) motility assay that determines the overall fitness of the worms, in terms of changes in motility, quantified as body bends per minute (BPM) and (ii) quantification of NIAD-4-stained Aβ42 aggregates (screening data shown in Supplementary Fig. 1). Panels **b**–**g** show characterization of kynurenic acid and carnosine to rescue a *C. elegans* model of AD. Panels (**b** and **c**) show the motility of worms, measured in body bends per minute (BPM) on *Y*-axis vs. days of adulthood on *X*-axis, treated with increasing concentrations of carnosine (blue) and kynurenic acid (red) compared to untreated worms (black). Increased thrashing frequency was observed across the lifespan of the worm up to 15 µM of carnosine and kynurenic acid (**b** and **c**). The motility at day 5 of adulthood, where phenotypic manifestations of Aβ42 are prominent in the GMC worm, is significantly improved by increasing carnosine up to 15 µM and for kynurenic acid at 10 µM (**d** and **e**). The radar chart shows the overall fitness of *C. elegans* as a function of speed, bends per minute (BPM) and the live ratio as is seen on each of its axis (**d** and **e**). Aggregate staining was quantified after worms were incubated with the amyloidogenic-specific dye NIAD-4 (**f**–**g**) (scale bar, 80 μm). White arrows in the panels (**f**–**g**) point to NIAD-4-stained Aβ42 aggregates, which appear orange-red in color. At all concentrations tested, carnosine treatment significantly inhibited Aβ42 aggregation compared to the GMC worms as shown in (**f**). N2 control worms, which do not express Aβ42, are shown for comparison (scale bar, 80 μm). Similar to carnosine, the aggregation of Aβ42 was inhibited by kynurenic acid treatment as shown in (**g**). For NIAD-4 screening of aggregates, approximately 15–23 animals were analyzed per condition for GMC (AD) worms and ∼10 animals per control (N2) worms. The beneficial effects of kynurenic acid and carnosine were observed in *n* = 3 biologically independent experiments. All error bars represent the standard error of the mean (SEM). Statistics were performed using one-way ANOVA, Dunnett’s multiple comparisons against the untreated Aβ42 group using GraphPad Prism, *p*-values are indicated on the plots.

*C. elegans* is a well-characterized model of aging sharing fundamental biochemical similarities with humans. Moreover, with an average lifespan of 2–3 weeks, it makes for a powerful in vivo tool for studying protein aggregation. Since we are investigating endogenous metabolites and wanted to investigate if and by which mechanism metabolites can clear aggregates, we considered selecting the *C. elegans* in vivo system for screening instead of an in vitro screening approach. We first carried out a dose-dependent screening (0, 1, 5, and 10 μM) (extended data in Supplementary Figs. 1 and 2) of the six metabolites that we identified as proteostasis regulators to test the overall fitness of a well-established *C. elegans* model, GMC101 (GMC), in which Aβ42 is overexpressed in the large muscle cells^39^. This model shows age-dependent inclusion formation and related toxicity, which can be measured by a decrease in the number of body bends per minute (BPM), an increase in paralysis rate, and a decrease in the speed of movement^39^ (Fig. 3). We report any significant benefits upon metabolite treatment as changes in overall fitness, where we consider fitness as the total behavioral response of worms as a function of BPM, speed (mm/s), and live ratio. We used Thioflavin-T (ThT) as a positive control which has been previously shown to profoundly extend lifespan and slow aging in adult *C. elegans*^40^; these beneficial effects of ThT depend on HSF-1, the stress resistance and longevity transcription factor SKN-1, molecular chaperones, autophagy, and proteasomal functions^40^ (Supplementary Figs. 1 and 2).

GMC and wild-type N2 worms were shifted onto the metabolites at the L4 stage, in order to avoid developmental effects, and subjected to motility assays on day 5 of adulthood, when the phenotypic manifestations of Aβ42 toxicity are prominent (Fig. 3). We found that three metabolites, carnosine, vanylglycol and kynurenic acid, improved the worm fitness as compared with the untreated GMC worms (Supplementary Figs. 1, 2, and for detailed characterization refer Fig. 3). We used ThT as a positive control for worm fitness^40^.   We found that the results obtained with carnosine and kynurenic acid were highly reproducible in terms of enhanced motility and markedly decreased NIAD-4-stained aggregates as compared to the untreated worms, and thus selected these two metabolites for further characterization.

At different doses of metabolites, both carnosine and kynurenic acid show a clear response in improvement of motility at day 5 (Fig. 3b–e) as compared with the initial (younger) days of adulthood. The GMC motility (in BPM) was highest at a concentration of 15 μM carnosine and 10 μM kynurenic acid, with a corresponding lower count of aggregates (Fig. 3f, g). We saw that carnosine is equally effective in the concentration range 10–25 μM, and. kynurenic acid was most effective in lowering aggregate number at a 15 μM concentration. In both cases, further increase in concentrations (≥50 μM) did not show any improvement in motility, possibly because of unrelated toxic effects of the metabolites in *C. elegans* (Supplementary Fig. 1h, i). We did not observe any overt  effects of carnosine and kynurenic acid on changes in worm motility and total fitness score in wild-type N2 worms (Supplementary Fig. 4). Total fitness score is calculated using metrics that include body bends, speed, paralysis rates, area per animal, and mean errors. The resulting fingerprint is generated in the form of a radar chart using the open-source software as described in Perni et al.^41^.

### Carnosine and kynurenic acid do not directly inhibit Aβ42 aggregation

**Fig. 4:**
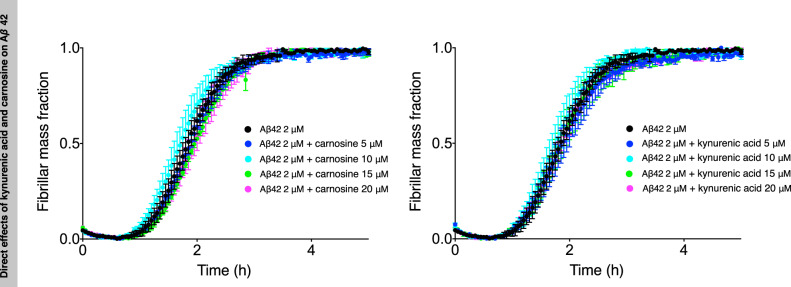
Carnosine and kynurenic acid do not have direct effects on Aβ42 aggregation. Kinetic profiles of the aggregation of a 2 μM Aβ42 sample in the absence (black) and the presence of increasing concentrations of carnosine or kynurenic acid (represented in different colors). The aggregation process of Aβ42 is not significantly accelerated or retarded by the presence of either of the metabolites (higher concentrations of the metabolites 20, 50, 100, and 500 μM are shown in Supplementary Fig. 3). Error bars are expressed as the standard deviations from three technical replicates.

To test whether carnosine or kynurenic acid could directly inhibit Aβ42 aggregation, we carried out a ThT fluorescence-binding chemical kinetics assay (see the “Methods” section). We did not observe, however, any direct effects of these two metabolites on the aggregation of Aβ42, at least over the relatively wide range of concentrations that we tested (Fig. 4 and Supplementary Fig. 3), which include physiologically relevant ones, indicating that another mechanism should be present that acts to clear the aggregates. Since the presence of this mechanism does not exclude the possibility that other endogenous metabolites may have a direct effect on Aβ42 aggregation, possibly by forming aggregates themselves or promoting phase separation of Aβ42 within the cytoplasm, we propose that it will be very interesting to test other endogenous metabolites for such a direct effect on protein aggregation.

### Carnosine and kynurenic acid increase levels of HSF-1 and molecular chaperones

**Fig. 5:**
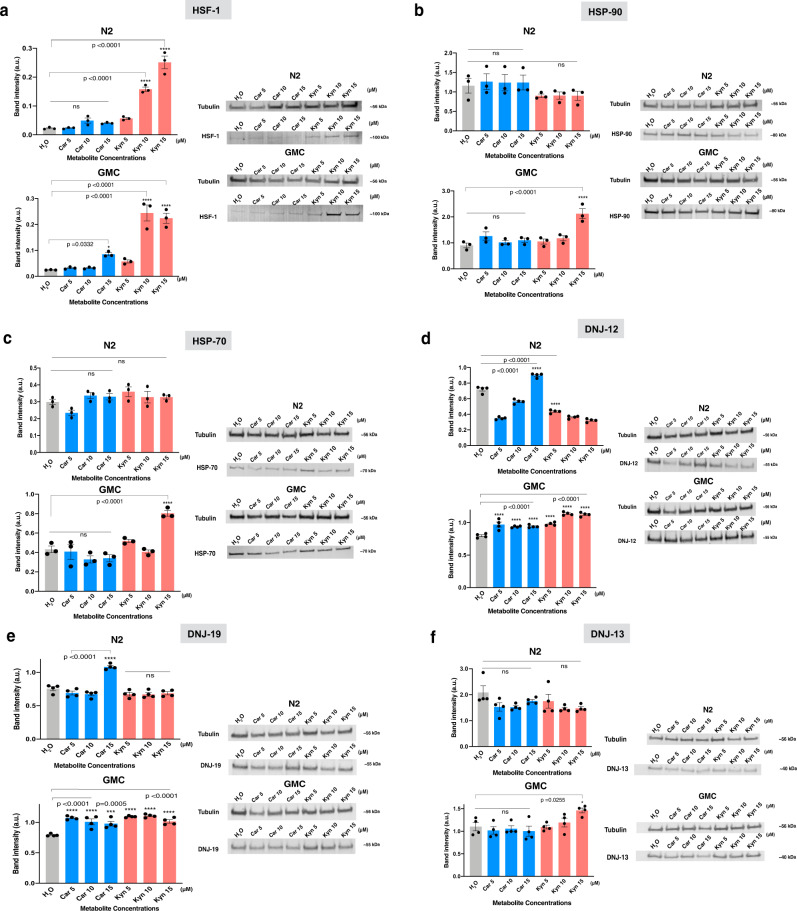
Carnosine and kynurenic acid activate a cytosolic unfolded protein response through an HSF-1-dependent mechanism. Western blots show a differential increase in protein levels on treatment with the two metabolites (**a**–**f**). On treatment with carnosine and kynurenic acid, we observe a relative increase in the protein levels of molecular chaperones and their co-chaperones in worm lysates relative to the controls, untreated N2 and GMC. *n* = ∼3000 worms per condition; we measured 3–4 replicates per condition; only a representative western blot is shown for each condition. From the experiments described in Fig. 3, a significant increase in motility and a corresponding decrease in the NIAD-4-stained aggregates were observed at a dose of 15 μM of both the metabolites. All error bars depict standard error of mean (SEM). **a**–**f** show bands of HSF-1, downstream molecular chaperones, and J proteins namely HSP-90, HSP-70, DNJ-12, DNJ-19, and DNJ-13, respectively, for both N2 (wild type) and GMC (AD model) worms. For each Western blot experiment, we used Tubulin signal to normalize for total protein concentration in each lane. We then normalized each condition of the chaperone/co-chaperone band with its corresponding tubulin band from the same experiment run on a parallel gel, to plot the intensities in ImageJ. The gels for each condition were run, respectively, at the same time, using the same running buffer, in the same electrophoretic cell and the same western blot transfer sandwich onto membranes. These membranes were further developed using appropriate antibodies (see the “Methods“ section, Supplementary Fig. 5). Statistics are performed in GraphPad Prism using ordinary one-way ANOVA; we used Dunnett’s multiple comparisons test with untreated (H2O) for each N2 and GMC metabolite-treated groups; *p*-values are indicated in the figure sub-panels.

We then investigated the effects of carnosine and kynurenic acid on the expression of HSF-1, the master regulator of the HSR in *C. elegans*. HSF-1 upregulates heat shock protein genes that act as molecular chaperones to maintain normal protein conformation under cellular stress, refold misfolded proteins, and target irreversibly damaged proteins for degradation^42–44^ (Fig. 5 and Supplementary Fig. 5).

We used an antibody validated to identify *C. elegans* HSF-1 by immunoprecipitation and mass spectrometry^45^, to probe for HSF-1 in lysates from worms treated with carnosine or kynurenic acid. We observed increased levels of HSF-1 following treatment with both the metabolites (Fig. 5a). Levels of HSF-1 increased in a dose-dependent manner in response to both carnosine and kynurenic acid treatment (Fig. 5a). In the *C. elegans* lysate treated with the two metabolites, we observed the HSF-1 band at ∼100 kDa.

HSF-1 primarily regulates the expression of genes encoding molecular chaperones, particularly the heat shock proteins HSP90, HSP70, and HSP40 that act as a cochaperone for HSP70^46–48^. We looked at the levels of core HSF-1-regulated chaperones and found that HSP90 and HSP70 levels were elevated in the GMC worms treated with 15 μM kynurenic acid, but not 15 μM carnosine as compared with untreated GMC and wild-type worms (Fig. 5b, c). Both DNJ-12 and DNJ-19 showed elevated levels in response to treatment with carnosine and kynurenic acid as compared to untreated GMC worms and treated wild-type N2 worms (Fig. 5d, e). In contrast, we did not observe significant changes in the levels of DNJ-13 upon metabolite treatment (Fig. 5f). DNJ-12 and DNJ-19 are class A J-protein (HSP40) cochaperones that have been characterized in vivo and in vitro for their protein disaggregase functions that promote organismal health^48^. J-proteins have also been previously characterized to have a disaggregase activity in a *C. elegans* polyQ model, where complexed J-protein co-chaperones of class A (DNJ-12 and DNJ-19) and B (DNJ-13) enable disaggregase activity through associations with HSP-110 and HSP-70, both individually and in a synergistic cooperation^48,49^. Our data suggest that an increase in HSF-1 levels upon treatment with carnosine and kynurenic acid promotes increased levels of molecular chaperones and cochaperones, in particular DNJ-12 and DNJ-19 to clear Aβ42 aggregates.

**Fig. 6:**
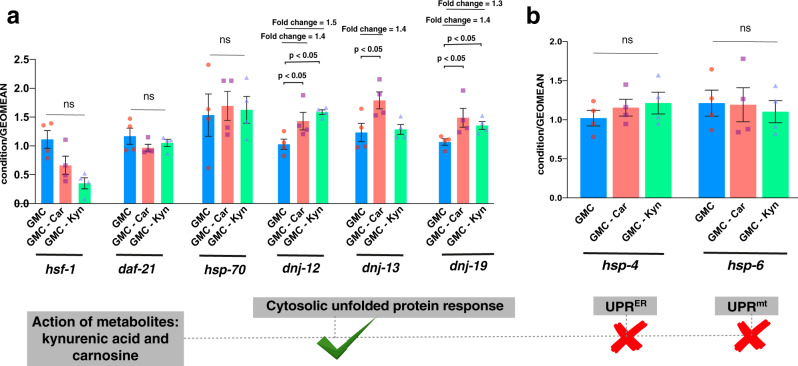
RTqPCR shows that carnosine and kynurenic acid activate a cytosolic unfolded protein response by elevating the transcript levels of the J-proteins *dnj*-12 and *dnj*-19 in day 5 GMC worms. GMC worms were administered 15 μM each of carnosine and kynurenic acid and their mRNA levels monitored by RTqPCR. **a** We did not observe any significant changes in the mRNA levels of *hsf-1*, *hsp-90*, and *hsp-70*. Carnosine treatment significantly elevated transcript levels of class A and B J-proteins, DNJ-12, DNJ-13, and DNJ-19. Kynurenic acid, however, elevated the levels only of class A J-proteins, DNJ-12, and DNJ-19. The changes in the transcript levels of J-proteins correlate to the protein level changes and are also supported by our RNAi knockdown experiments, where DNJ-13 does not show a role in clearing out the aggregates of Aβ42 aggregates (Fig. 8a, b). **b** We also tested for any changes in the UPR^ER^ and UPR^mt^ after giving carnosine and kynurenic acid, however, found no significant changes in the markers, *hsp-4* and *hsp-6*, corresponding to the activation of these pathways, respectively. All data represent four biological replicates of day 5 *C. elegans* GMC. Y-axis represents condition/GEOMEAN where condition refers to each *hsf-1, daf-21, hsp-70, dnj-12, dnj-13, dnj-19, hsp-4,* and *hsp-6*, and GEOMEAN is the geometric mean of two housekeeping genes *rpb-2* and *cdc-42* for normalization. We performed statistics using GraphPad Prism using one-way ANOVA with Tukey pairwise comparison of columns against the untreated GMC (AD) worms for each condition treated with metabolites. All error bars represent standard error of mean (SEM). Significance *p*-values are indicated above the bar plot. ns indicates statistically non-significant.

We then asked whether the changes in protein levels were due to altered transcription by performing real-time quantitative PCR (RTqPCR). No significant fold changes in the mRNA levels of *hsf*-1, *daf-21,* and *hsp*-70 were observed following treatment with either metabolite. However, consistent with our protein data, we observed that the mRNA levels of *dnj*-12 and *dnj*-19 were elevated by treatment with both metabolites. Intriguingly, the mRNA levels for *dnj*-13 were also elevated in carnosine, but not kynurenic acid, treated worms, despite the fact that no significant changes were observed at the protein level (Fig. 6a).

We further asked if these metabolites triggered UPR related to the ER (UPR^ER^) or mitochondria (UPR^mt^) by probing for mRNA levels of *hsp-4* and *hsp-6*, the canonical markers of the UPR^ER^ and UPR^mt^, respectively^50,51^. There were no significant changes in these two markers compared with the controls (Fig. 6b), thus excluding these responses as underlying mechanisms for clearance of aggregates in this study.

**Fig. 7:**
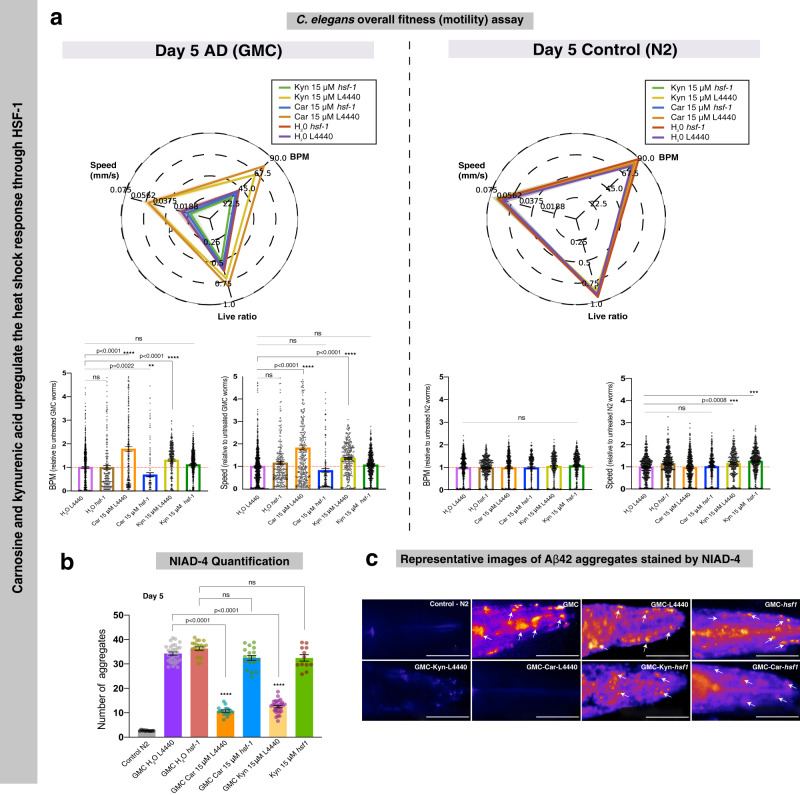
Carnosine and kynurenic acid protect against Aβ42 aggregation and toxicity in an HSF-1-dependent manner. **a** RNAi knockdown of *hsf-1* shows decreased motility in GMC (AD) worms. L4440 is the empty vector control. On treatment with carnosine and kynurenic acid, a significant increase in motility is observed in the L4440 phenotype as compared to a decrease in the *hsf-1(RNAi)* groups. The control N2 worms show no significant effect of the metabolites carnosine (Car) and kynurenic acid (Kyn). The radar chart depicts overall fitness by comparing the three quantitative variables: worm speed (mm/s), bends per minute and live ratio as is seen on its axis. The bar graph inset shows that RNAi knockdown of *hsf-1* significantly impairs the beneficial effects of carnosine and kynurenic acid. Statistics were performed using one-way ANOVA, Dunnett’s multiple comparisons test against the untreated Aβ42 empty vector. ****p < 0.0001; ***p < 0.001; **p < 0.01; *p < 0.05 (**b**). A corresponding decrease in NIAD-4 aggregates is observed in AD worms treated with carnosine and kynurenic acid (Scale bars, 80 μm). For NIAD-4 staining, approximately 11–30 animals were analyzed per condition. All error bars represent SEM. Statistics were performed using one-way ANOVA Dunnett’s multiple comparisons test against the untreated Aβ42 empty vector. ****p < 0.0001; ***p < 0.001; **p < 0.01; *p < 0.05. **c** Representative images of Aβ42 plaques show a significant clearance of aggregates in worms treated with carnosine (Car) and kynurenic acid (Kyn). *hsf-1* knockdown worms even when treated with carnosine and kynurenic acid did not clear aggregates. White arrows point to NIAD-4-stained Aβ42 aggregates, which appear orange-red in color. For comparison, an empty RNAi vector L4440 GMC worm head is shown.

To determine whether carnosine and kynurenic acid treatment suppress Aβ42 toxicity and aggregation through HSF-1 and increased levels of molecular chaperones, we used RNA interference (RNAi) to knockdown specific genes and asked whether this abrogated the effects of carnosine or kynurenic acid on Aβ42 aggregation and toxicity. If HSF-1 is playing a role in the clearance of Aβ42 aggregates, then HSF-1 knockdown would show an obvious aggregate-positive phenotype, with heightened worm paralysis. Indeed, this was the case when we performed RNAi against *hsf-1* (see the “Methods” section) and treated the GMC worms with water, carnosine, and kynurenic acid (Fig. 7). In an empty vector L4440, we see that both carnosine and kynurenic acid clear Aβ42 aggregates and increase overall fitness when compared with the RNAi worms where *hsf-1* is knocked down prior to treatment with the two metabolites (Fig. 7a). In these GMC *hsf-1* knockdown worms, we see a higher number of Aβ42 aggregates (Fig. 7b, c), together with a decrease in overall fitness (Fig. 7a), even when supplemented with the metabolites. Thus, overall, we see that the beneficial effects of carnosine and kynurenic acid were markedly reduced after knocking down *hsf-1* using RNAi (Fig. 7a). Note that the wild-type N2 worms, both treated and untreated, showed no overt differences in overall paralysis on *hsf-1* knockdown (Fig. 7a radar plot and bar plots N2). Previous studies have shown that the complete effect of RNAi can take over 48 h to establish a significant knockdown phenotype^52,53^, hence at day 5, our results are in agreement. As a further study, it will be interesting to explore the effect of these metabolites on the time course of ageing in healthy worms.

**Fig. 8:**
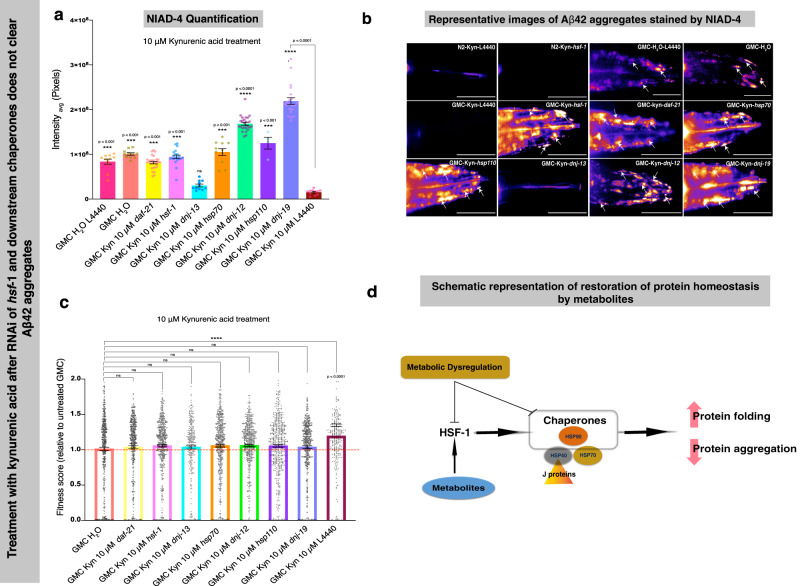
Kynurenic acid prevents Aβ42 toxicity in a *C. elegans* model of AD through the action of molecular chaperones. On treatment with 10 μM kynurenic acid, a cytosolic unfolded protein response is activated by HSF-1 and involves HSP-90, HSP-70, HSP-110, HSP40 J proteins DNJ-12 and DNJ-19, but not DNJ-13 (panels **a**–**c**). The  overall fitness (bends per minute, speed and survival) in a *C. elegans* model of AD (GMC worms) is compromised even after treatment with 10 μM kynurenic acid in the *hsf-1, daf-21, hsp-70, hsp-110, dnj-12, dnj-13, and dnj-19* knockdowns as compared to the control L4440 (**c**). A corresponding increase in NIAD-4 aggregates is observed (**a** and **b**). However, we do not see any aggregates on knocking down *dnj*-13, a class B J-protein, suggesting it may not play a role in clearing out Aβ42 aggregates. The white arrows point to NIAD-4-stained Aβ42 aggregates, which appear orange-red in color (scale bars, 80 μm). For motility assays *n* = ∼260–1000 animals per condition, for NIAD-4 staining assay *n* = 11–29 for all conditions except *hsp110 knockdown*, for which *n* = 4. All error bars represent SEM. **a** and **c** statistics were preformed using one-way ANOVA, Dunnett’s multiple comparisons test against the control L4440 AD group treated with 10 μM kynurenic acid, ****p < 0.0001; ***p < 0.001; **p < 0.01; *p < 0.05. **d** Schematic of the mechanism by which the metabolites clear protein aggregates by mediating a heat shock response. We propose that metabolic dysregulation may thus be a prior contributory factor to an imbalance in protein homeostasis.

Next, we individually knocked down the genes *daf-21* (HSP90), *hsp-1* (HSP70), *dnj-13*, *dnj-12*, *dnj-19,* and *hsp-110* using RNAi to study the consequent effects on *C. elegans* motility after treatment with kynurenic acid. DNAJ (HSP40) interacts with HSP70 to promote refolding and HSP110 to promote disaggregation. Therefore, we also considered HSP110 in our RNAi study to check for overall behavioral effects and Aβ42 aggregation in *C. elegans*. We observed increased Aβ42 aggregates in each knockdown GMC group that was otherwise suppressed by treatment with kynurenic acid (Fig. 8a, b). Although we observed no significant effects in each knockdown condition (Fig. 8c) at day 5 of adulthood after kynurenic acid treatment, there was a significant increase in overall fitness in the empty vector L4440 upon kynurenic acid treatment  as compared with the untreated GMC (AD) worms. This shows that kynurenic acid indeed clears Aβ42 aggregates, which is mediated by these chaperones, thus resulting in an improvement in the motility of Aβ42-expressing worms.

Taken together, our data from western blots, RTqPCR, and RNAi suggests that treatment with kynurenic acid and carnosine elevates the levels of HSF-1 in a *C. elegans* model of AD, resulting in transcriptional upregulation and thus increased levels of DNJ-12 and DNJ-19 J-proteins. This then appears to cooperate with and at least requires multiple core chaperones such as HSP70, HSP90, and HSP110 to protect against Aβ42 aggregation and associated toxicity in *C. elegans* (Fig. 8c).

Together, our data show how two metabolites trigger a cytosolic unfolded protein response in a *C. elegans* model of AD to clear Aβ42 aggregates and their associated toxicity. Since levels of these metabolites may be age-related or disease-related, we propose that metabolites can be inducers of protective quality control cellular responses that protect against disease-associated protein aggregation (Fig. 8c).

## Discussion

We have shown how the protein homeostasis network, which comprises several interdependent mechanisms including molecular chaperones, detoxifying enzymes, and protein clearance mechanisms^54^, can be modulated by endogenous metabolites. We have reported a library of endogenous metabolites that can be further explored for action on various cellular mechanisms associated with protein homeostasis, and we characterized two metabolites, carnosine and kynurenic acid, for their role in the clearance of protein aggregates. These two metabolites are able to inhibit Aβ42 aggregation in a *C. elegans* model of AD by triggering a cytosolic unfolded protein response mediated through HSF-1 and increased levels of the DNJ-12 and DNJ-19 J-proteins. We anticipate that further studies will reveal how these metabolites increase the levels of HSF-1 and thereby regulate its function.

We propose a model for the restoration of protein homeostasis by endogenous metabolites (Fig. 8c), where the two endogenous metabolites regulate HSF-1 and downstream chaperones to clear Aβ42 aggregates. With regard to clearance of aggregates, it is plausible that (1) the aggregates are removed en masse by autophagy, as autophagy has been linked to HSF-1 activity by Kumsta and coworkers^55^, or (2) increased chaperones promote the turnover of Aβ42 protein, thereby suppressing aggregate formation. It cannot be that chaperones are just blocking aggregation as we do not see a corresponding increase in soluble Aβ42 levels.

Carnosine is a dipeptide that occurs naturally in the brain, kidney, and skeletal muscle of fish, birds, and mammals^56^. Post-mitotic adult cells have higher carnosine concentrations than actively dividing cells, although the reasons are not clear^57,58^; carnosine synthesis is only associated with final stages of glial cell maturation. It is also present only in post-mitotic retinal neurons when energy metabolism switches from glycolysis to oxidative phosphorylation^56^. Interestingly, carnosine has also been observed to have a beneficial but unspecified organizational effect towards mitochondria; its levels change with respect to energy metabolism of the cell in ageing^56^. Taken together, these studies suggest that carnosine has beneficial effects on cellular activity. We see that in disease carnosine is downregulated (Table 1), and our studies indicate that supplementation rescues AD disease phenotype. Its subsequent metabolism on supplementation is a problem underscored for future study. Elucidation of its mechanism of action in the context of clearing protein aggregates, therefore, is useful in exploiting its therapeutic potential.

Kynurenic acid is a tryptophan metabolite, which has anti-inflammatory and immunosuppressive functions^59,60^, in addition to acting as an antagonist affecting all ionotropic glutamate receptors including NMDA, AMPA, and kainite receptors^61^. Although kynurenic acid has been implicated to be neuroprotective in a vast majority of neurological conditions, its mechanisms of action have not been fully clarified. Kynurenic acid and associated metabolite levels are reported to be dysregulated in people with sleep disorders^62,63^ and depression^64^ showing both upregulation and downregulation (Table 1). Sleep/wake patterns have been reported to be disrupted in AD^65^. Although depression has been associated as a risk factor for developing AD, a genome-wide association study indicated no significant genetic overlap between the two diseases^66^; interestingly, the overlap could indeed be explained by a disruption of neurotransmitter homeostasis^67^. Further, endurance exercise has been reported to increase plasma levels of kynurenic acid^68 ^. Previously, kynurenine pathway has been suggested to be a regulator of age-related protein toxicity^69^. In a *C. elegans* model, van der Goot et al. show that depletion of tryptophan 2,3-dioxygenase (*tdo-2*), the first enzyme in the kynurenine pathway of tryptophan degradation, increases tryptophan levels and suppresses toxicity of aggregation-prone proteins; moreover, they show that feeding L-tryptophan also suppresses toxicity^69^.    In this study, we show a rescue of AD disease phenotype by kynurenic acid supplementation; it is known that in *C. elegans* and mammals, kynurenic acid is generated in spatially restricted patterns in the nervous system, so we think that modifying its levels endogenously, in a tissue-specific manner, will further give insights into how its endogenous levels affect target proteins. Our results on its mechanism of action on clearing protein aggregates shed light on systematically exploiting its therapeutic potential.

Taken together, our results emphasize the need to explore in more detail the effect of abnormal metabolite homeostasis in the development of protein misfolding disorders.

## Conclusions

The results that we have reported in this study support the view that metabolite homeostasis and protein homeostasis are closely linked and essential for overall cellular homeostasis. In particular, metabolite homeostasis can be disrupted in an age-related manner and may be associated with protein misfolding diseases. In general, our results support the view that rebalancing cellular homeostasis through the maintenance of metabolite homeostasis may offer preventative approaches for neurodegenerative diseases.

## Methods

### Identification of endogenous metabolites in the HMDB

We used 200 molecules as starting points, hereby referred to as seeds, from Calamini et al. ^34^ to calculate fragments using a method reported by Joshi et al. ^35^. We screened the HMDB using similarity and Tanimoto coefficients (>0.6) of the initial seeds. The hits obtained were filtered if they satisfied the following conditions: endogenous, quantified and detected, detected in the CSF. We further calculated if they were upregulated or downregulated based on the normal vs. abnormal physiological concentrations reported in the HMDB. This step was performed by literature search to assess their association with disease, where we ranked the metabolites based on an association score, which we operationally defined as the number of studies in which a metabolite was reported as dysregulated in AD. To this end, we searched in PubMed “Alzheimer’s disease AND metabolite”, where “metabolite” refers to each metabolite in Supplementary Data 1.

### Chemicals used in this study

All metabolites: kynurenic acid (K3375) carnosine (C9625), melatonin (M5250), 1-methylhistidine (67520), homovanillic acid (69673) were purchased from Sigma-Aldrich (Germany). Vanylglycol was purchased from MolPort (MolPort-022-375-161). All metabolites were dissolved in water.

### Expression, purification, and preparation of Aβ42 peptide samples for kinetic experiments

The recombinant Aβ42 peptide (MDAEFRHDSGYEVHHQKLVFFAEDVGSNKGA IIGLMVGGVVIA) was expressed in the *Escherichia coli* BL21 Gold (DE3) strain (Stratagene, CA, USA) and purified as described previously^70^. Briefly, in the purification procedure, the *E. coli* cells were sonicated, and the inclusion bodies were subsequently dissolved in 8 M urea. A diethyl-aminoethyl cellulose resin was then used to perform ion-exchange chromatography, and the protein collected was lyophilized. These fractions were then further purified using a Superdex 75 26/60 column (GE healthcare, IL, USA), and the fractions containing the recombinant protein were combined, frozen, and lyophilized again. Solutions of monomeric protein were prepared by dissolving the lyophilized Aβ42 peptide in 6 M GuHCl, and purified in 20 mM sodium phosphate buffer, 200 µM EDTA, pH 8.0 using a Superdex 75 10/300 column (GE Healthcare) at a flow rate of 0.5 ml/min. ThT was added from a 2 mM stock to give a final concentration of 20 µM. All protein samples were obtained from the same single preparation and then pipetted in low-binding Eppendorf tubes. Each sample was then pipetted into multiple wells of a 96-well half-area, low-binding, clear bottom and PEG coating plate (Corning 3881), 80 µl per well, in the absence and the presence of different molar-equivalents of carnosine or kynurenic acid, to give a final concentration of 1% DMSO (v/v).

The aggregation of Aβ42 (at 2 µM concentration^70,71,72^, was initiated by placing the 96-well plate in a plate reader (Fluostar Omega or Fluostar Optima from BMG Labtech, Aylesbury, UK) at 37 °C under quiescent conditions. The ThT fluorescence was monitored in triplicate per sample as measured using bottom-optics with 440 nm excitation and 480 nm emission filters. Kinetic traces were then obtained for each well, and then processed to the fibrillar mass fraction by normalizing the ThT fluorescence values at a given time *t* against the initial ThT fluorescence value (which is taken as 0), and the final ThT fluorescence value at the end of the reaction (which is taken as 1.0).

### *C. elegans* experiments

#### Media

Standard conditions were used for the propagation of *C. elegans*^73^. Briefly, animals were synchronized by hypochlorite bleaching, hatched overnight in M9 (3 g/l KH_2_PO_4_, 6 g/l Na_2_HPO_4_, 5 g/l NaCl, 1 µM MgSO_4_) buffer, and subsequently cultured at 20 °C on nematode growth medium (NGM) (CaCl_2_ 1 mM, MgSO_4_ 1 mM, cholesterol 5 µg/ml, 250 µM KH_2_PO_4_ pH 6, Agar 17 g/l, NaCl 3 g/l, casein 7.5 g/l) plates seeded with the *E. coli* strain OP50. Saturated cultures of OP50 were grown by inoculating 50 ml of LB medium (tryptone 10 g/l, NaCl 10 g/l, yeast extract 5 g/l) with OP50 and incubating the culture for 16 h at 37 °C. NGM plates were seeded with bacteria by adding 350 µl of saturated OP50 to each plate and leaving the plates at 20 °C for 2–3 days. On day 3 after synchronization, the animals were placed on NGM plates containing 5-fluoro-2′deoxy-uridine (FUDR) (75 µM) to inhibit the growth of offspring and the temperature was raised to 24 °C.

#### Strains

All strains were acquired from the Caenorhabditis Genetics Center in Minnesota, which is supported by NIH P40 OD010440. Two strains were utilized for these experiments. The temperature sensitive human Aβ-expressing strain dvIs100 [unc-54p::A-beta-1-42::unc-54 3′-UTR + mtl-2p::GFP] (GMC101) was used, in which mtl-2p::GFP causes intestinal GFP expression and unc-54p::A-beta-1-42 expresses the human full-length Aβ_42_ peptide in the muscle cells of the body wall. Raising the temperature above 20 °C at the L4 or adult stage causes paralysis due to Aβ_42_ aggregation in the body wall muscle. The N2 strain was used for wild-type worms.

#### Metabolite-coated plates

Aliquots of NGM media were autoclaved and poured and seeded with 350 µl OP50 culture and grown overnight. After incubating for up to 3 days at room temperature, aliquots of l-carnosine (carnosine) or kynurenic acid (or other metabolites) dissolved in water at different concentrations were added. NGM plates containing FUDR (75 µM, unless stated otherwise) were seeded with 2.2 ml aliquots of compound dissolved in water at the appropriate concentration. The plates were then placed in a laminar flow hood at room temperature to dry and the worms were transferred to plates coated with metabolite at larval stage L4. The six metabolites were initially screened in liquid media^41^. Carnosine was prepared at room temperature to a stock concentration of 5 mM. Kynurenic acid was prepared at 5 mM and compound dissolution was carried out at 100 °C. Stocks of the compound were maintained at −20 °C until use and never thawed more than once.

#### Automated motility assay

At different ages, animals were washed off the plates with M9 buffer and spread over an OP50 un-seeded 6 cm plate, after which their movements were recorded at 20 fps using a recently developed microscopic procedure for 60–90 s^41,74^. Approximately 100–600 animals were counted per condition at each indicated timepoint, unless otherwise specified for >600 (Supplementary Data 2). The automated motility tracker software avoids the underestimation of errors resulting from worm collisions and overlap; it detects the total number of worms, and also provides the upper limit on errors by considering the maximum number of worms present in a single frame at the same time (Supplementary Data 2)^41^.

#### NIAD-4 staining and imaging

NIAD-4 solution was prepared by dissolution in 100% DMSO at 1 mg/ml. Prior to worm incubation, a 1/1000 dilution in M9 was created. After screening using the Wide-field Nematode Tracking Platform, ~300 worms per condition were collected in M9 media and centrifuged at 20 °C at 2000 rpm for 2 min to a pellet. 1 ml of diluted NIAD-4 solution in M9 was then added to the pellet and placed under gentle shaking (80 rpm) for 6 h. Worms were then transferred to unseeded NGM plates and incubated at 20 °C for twelve hours. Worms were again washed from the plates with M9 media, spun down, washed with 10 ml M9, and resuspended in 2 ml M9. After gravity sedimentation, 15 µl worm solution was spotted on 4% agarose pads. To anaesthetize the animals, 4 µl of 40 mM NaN_3_ was added, followed by a glass coverslip. Worms were imaged using a Zeiss Axio Observer A1 fluorescence microscope (Carl Zeiss Microscopy GmbH, Jena, Germany) with a ×20 objective and a 49004 ET-CY3/TRITC filter (Chroma Technology Corp, VT, USA). An exposure time of 1000 ms was employed. The nominal magnification was ×40 and images were captured using an Evolve 512 Delta EMCCD camera with a high quantum efficiency (Photometrics, Tucson, AZ, USA). Approximately 11–30 animals were analyzed per condition, unless otherwise specified, and statistics were performed using the one-way ANOVA against the untreated group. All statistics herein were performed using GraphPad Prism. Quantification was performed using ImageJ (NIH, MD, USA) to determine the grayscale intensity mean in the head of each animal. The “fire” filter was used in ImageJ for visualization of NIAD-4-stained aggregates. Total intensity (mean gray value) of the worm head was calculated by outlining the worm head area from the nose to the pharynx. At day 5, the maximum diameter of the worms across the head is 80 µm. Then the average (three readings taken) background of the same area elsewhere on the image was calculated and subtracted from the total mean gray value of the worm head. The resultant intensity, after background subtraction, gives a readout of the NIAD-4 stained aggregates, which appear orange-red and are marked with arrows in relevant figure panels.

### Western blots

Approximately 3000 worms per condition were lysed in PBS. A cocktail of protease inhibitor was added to the lysate (Sigma MS-SAFE Protease and Phosphatase Inhibitor Cocktail). Anti-tubulin (Monoclonal anti-alpha-Tubulin antibody produced in mouse, Sigma, T6074) was used for detection of constitutively expressed tubulin for normalization of protein concentrations. HSF1 antibody was procured from Veena Prahlad (Iowa), HSP90 (rabbit anti-DAF-21) from Patricia Van Oosten-Hawle (Leeds), HSP40 (anti-DNJ-12, DNJ-13, and DNJ-19, all raised in rabbits) from Janine Kirstein (Berlin), HSP70 from John Labbadia (UCL, London). Mouse and rabbit secondary antibodies were used (Alexa Fluor 488-conjugated secondary antibodies). All quantifications were performed using ImageJ software to determine the protein band intensity using densitometry. 3-4 technical replicates were used per condition and statistics were performed using the two-way ANOVA against the untreated wild type (N2) and treated Aβ42 (GMC) worms groups. All statistics were performed using GraphPad Prism.

### RNA interference

Bacteria harboring an empty vector (L4440) or a vector containing *hsf-1* dsRNA, were grown at 37 °C overnight in LB containing 100 μg/ml ampicillin. Following overnight growth, cultures were induced with 5 mM IPTG and grown for a further 3 h at 37 °C. RNAi cultures were allowed to cool to room temperature and then seeded onto NGM plates containing 100 μg/ml ampicillin and 1 mM IPTG. Bacterial lawns were then left to dry for 4 days on the bench. The control, *hsf-1* and downstream chaperone RNAi clones were obtained from the Ahringer RNAi library and were sequence validated before use. Futhermore, these clones were shown to knock-down their intended targets in the previous publications^48,75,76^.

### RNA extractions, cDNA synthesis, and RTqPCR

Approximately 700, day 5 adult worms, were resuspended in 250 μl Trizol and homogenized by vortexing three times for 10 min continuously, with 20 min rest periods on ice between each round of vortexing. Chloroform was added (1/5th volume) before samples were shaken for 15 s by hand, and then centrifuged at 13,000×*g* for 15 min. The aqueous phase was collected and mixed with an equal volume of 70% ethanol. RNA was then purified using an RNeasy kit as per manufacturer’s instructions. cDNA was then synthesized from 1 µg total RNA using iScript cDNA synthesis reagents (BioRad).

RTqPCR was performed using BioRad Advanced SYBR green master mix and a BioRad CFX96 thermocycler using primers described in Table 2. The standard curve method was used to quantify gene expression and relative-expression of genes of interest was normalized to the house-keeping genes *rpb-2* and *cdc-42*.

**Table 2:** Primers (5′–3′) used for real-time quantitative PCR.

*gene*	Forward	Reverse
*rpb-2*	AACTGGTATTGTGGATCAGGTG	TTTGACCGTGTCGAGATGC
*cdc-42*	TCGACAATTACGCCGTCACA	GAAACACGTCGGTCTGTGGA
*hsf-1*	GGACACAAATGGGCTCAATG	CGCAAAAGTCTATTTCCAGCAC
*hsp-70*	CTACATGCAAAGCGATTGGA	GGCGTAGTCTTGTTCCCTTC
*hsp-90*	GACCAGAAACCCAGACGATATC	GAAGAGCACGGAATTCAAGTTG
*dnj-12*	GGTTCAACAAATGCAATCTCACT	CGTCTTCTTTCACCTGCTTCT
*dnj-13*	ACGATGTCATTAAGCCGGG	GAGTTGGATTGAGTTGTGATGG
*dnj-19*	GTGAGCCAGGAGATGTTGTC	CAGTGATAGTTTCTTGGTCATGTG
*hsp-4*	GGGGACAATCATTGGTATCG	ACGCAACGTATGATGGAGTG
*hsp-6*	AACCATTGAGCCATGCCGTA	CTTGAACAGTGGCTTGCACC

### Statistics and reproducibility

The data presented as mean ± SEM were tested for significance in the one-way ANOVA, using GraphPad Prism. Post-hoc comparisons were conducted using Dunnett’s multiple comparisons (Figs. 3, 5, 7, and 8) and Tukey’s pairwise comparison (Fig. 6). For kinetic profiles of aggregation in Fig. 4, error bars are represented from three technical replicates. Sample size: (a) motility assays *n* = 100–600 worms, unless otherwise specified (b) western blots *n* = ∼3000 worms for each experimental replicate, (c) NIAD-4 aggregation assay *n* = 11–30 animals per condition for GMC and *n* = ∼10–12 animals per control (N2), and (d) qPCR *n* = 4 biological replicates. All screenings, including RNAi, were conducted under worm-motility tracker  blinding to group identity, and then  matched. Significant results were marked according to critical *p*-values: ****p < 0.0001; ***p < 0.001; **p < 0.01; *p < 0.05.

### Reporting summary

Further information on research design is available in the Nature Research Reporting Summary linked to this article.

## Supplementary information

Supplementary Information

Description of Additional Supplementary Files

Supplementary Data 1

Supplementary Data 2

Reporting Summary

## Data Availability

All data supporting the findings of this study are included in the main article and its supplementary information files (Supplementary information, Supplementary Data 1 and Supplementary Data 2). Raw data are also available from the corresponding author(s) upon reasonable request.
