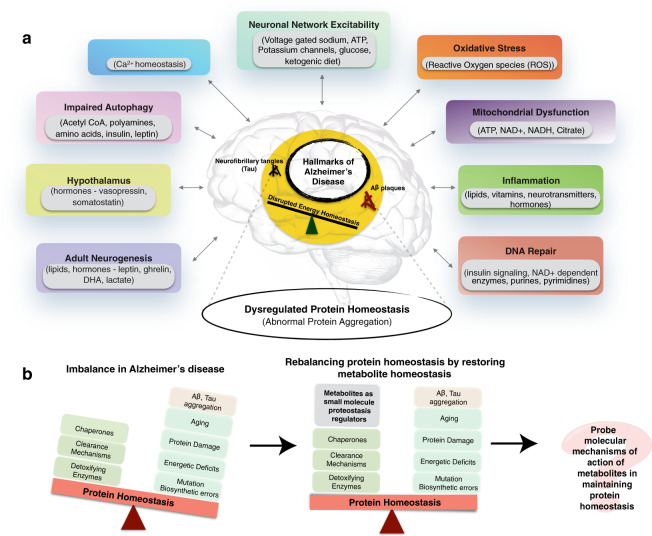# Publisher Correction: Two human metabolites rescue a *C. elegans* model of Alzheimer’s disease via a cytosolic unfolded protein response

**DOI:** 10.1038/s42003-021-02457-8

**Published:** 2021-07-26

**Authors:** Priyanka Joshi, Michele Perni, Ryan Limbocker, Benedetta Mannini, Sam Casford, Sean Chia, Johnny Habchi, Johnathan Labbadia, Christopher M. Dobson, Michele Vendruscolo

**Affiliations:** 1 Yusuf Hamied Department of Chemistry, Centre for Misfolding Diseases, University of Cambridge, Cambridge, UK; 2 Department of Genetics, Evolution and Environment, Institute of Healthy Ageing, University College London, London, UK; 3 Present Address: The California Institute for Quantitative Biosciences (QB3-Berkeley), University of California, Berkeley, CA USA; 4 Present Address: Department of Chemistry and Life Science, United States Military Academy, West Point, NY USA

**Keywords:** Molecular biology, Neuroscience

Correction to: *Communications Biology* 10.1038/s42003-021-02218-7, published online 7 July 2021.

In the original published version of the Article, Fig. 1 contained errors affecting several of the text labels. The original and corrected versions of the figure are shown below. The errors have been corrected in the HTML and PDF versions of the Article.

Original published version:


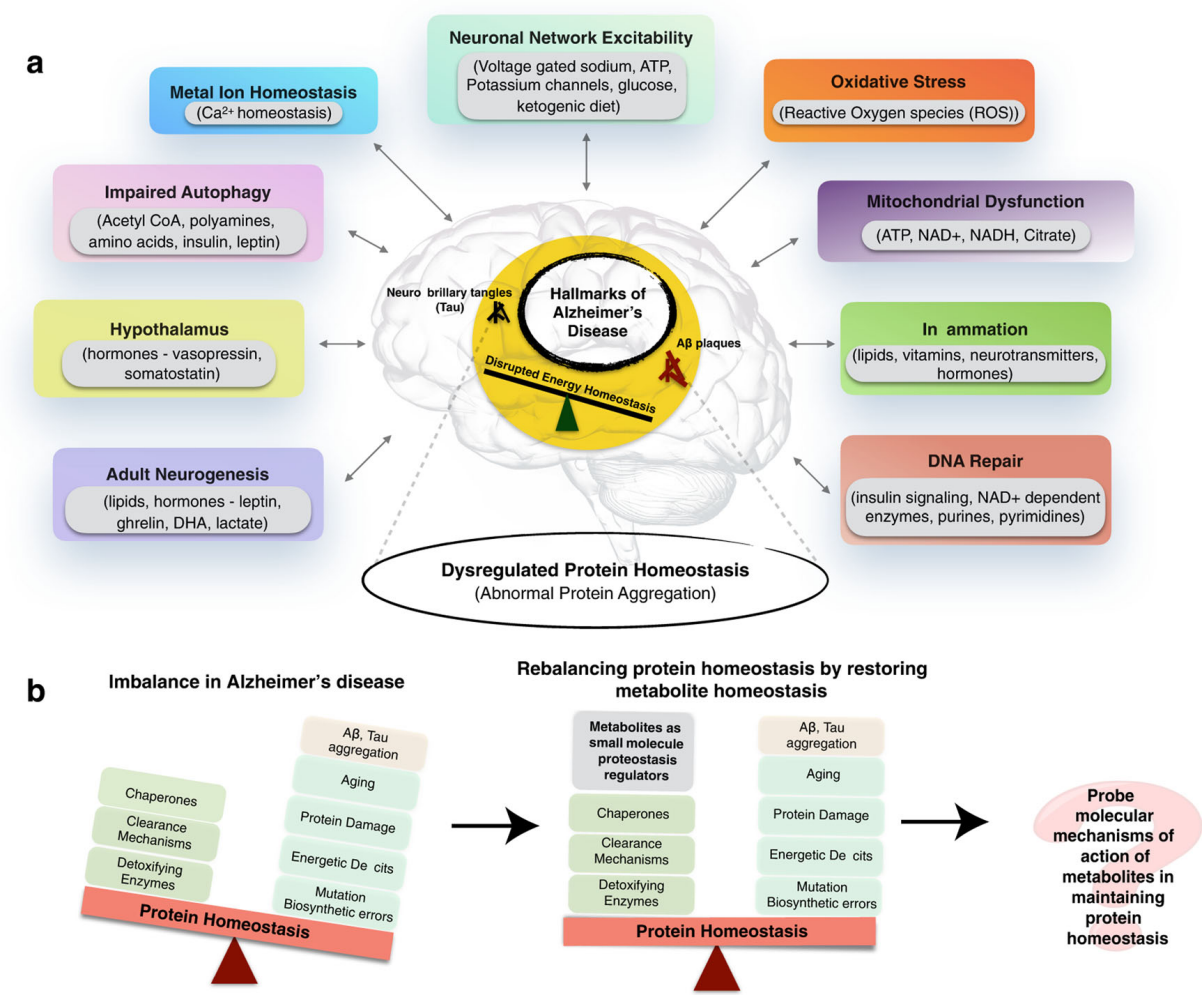


Corrected version: