# Supplementary material for: Two human metabolites rescue a C. elegans model of Alzheimer’s disease via a cytosolic unfolded protein response
Source: Commun Biol. 2021 Jul 7;4:843. doi: 10.1038/s42003-021-02218-7 (PMC8263720; doi:10.1038/s42003-021-02218-7)
Supplement: Supplementary file 1 — Supplementary Information [file 42003_2021_2218_MOESM1_ESM.pdf]

Two human metabolites rescue a *C. elegans* model of Alzheimer's disease via a cytosolic unfolded protein response

Priyanka Joshi[1,4,*], Michele Perni[1], Ryan Limbocker[1,3], Benedetta Mannini[1], Sam Casford[1], Sean Chia[1], Johnny Habchi[1], Johnathan Labbadia[2], Christopher M Dobson[1] and Michele Vendruscolo[1,*]

[1]Centre for Misfolding Diseases, Yusuf Hamied Department of Chemistry, University of Cambridge, Cambridge CB2 1EW, UK

[2]Institute of Healthy Ageing, Department of Genetics, Evolution and Environment, University College London, London WC1E 6BT, UK

[3]Current address: Department of Chemistry and Life Science, United States Military Academy, West Point, NY 10996, USA

[4]Current address: The California Institute for Quantitative Biosciences (QB3-Berkeley), University of California, Berkeley, CA 94720, USA

Supplementary Information

*Correspondence to: prijoshi@berkeley.edu, mv245@cam.ac.uk

Figure S1. (**a-f**) Effects of the six metabolites identified in this work on *C. elegans* GMC motility at various stages (day 1, 3, 4 and 5) of adulthood at concentrations 0, 1, 5 and 10 µM. (**g**) Effects of metabolites at day 5 of adulthood at 10 µM metabolites or 200 µM ThT. (**h-i**) Extended data from Figure 3(d-e) shows a dose-dependent effect (at concentrations 5, 10, 15 and 25 µM) of carnosine and kynurenic acid treatment on *C. elegans* GMC motility at day 5 of adulthood. Error bars indicate SEM. Statistics were preformed using one-way ANOVA, Dunnett's multiple comparisons against the untreated Aβ42 group using GraphPad Prism; ****, $p<0.0001$; ***, $p<0.001$; **, $p<0.01$; *, $p<0.05$).

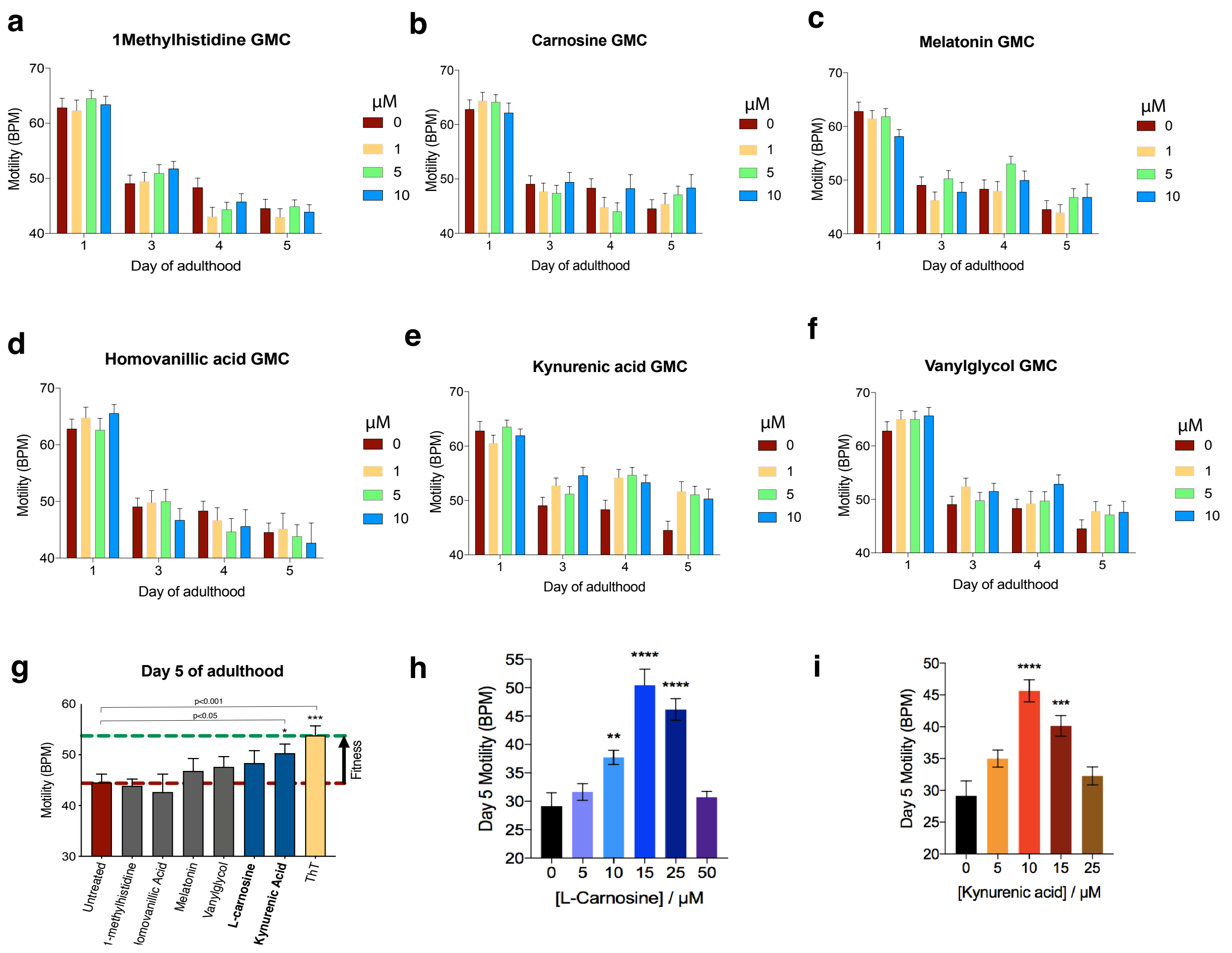

Figure S2. The amyloid-binding dye thioflavin T (ThT) increases motility in *C. elegans* by reducing Aβ42-associated toxicity. ThT increases lifespan in *C. elegans* by reducing Aβ42-associated toxicity [1]. Here, we see a dose-dependent increase in worm motility (0, 50, 100 and 200 μM) at day 5. X-axis depicts days of adulthood and y axis bends per minute (BPM).

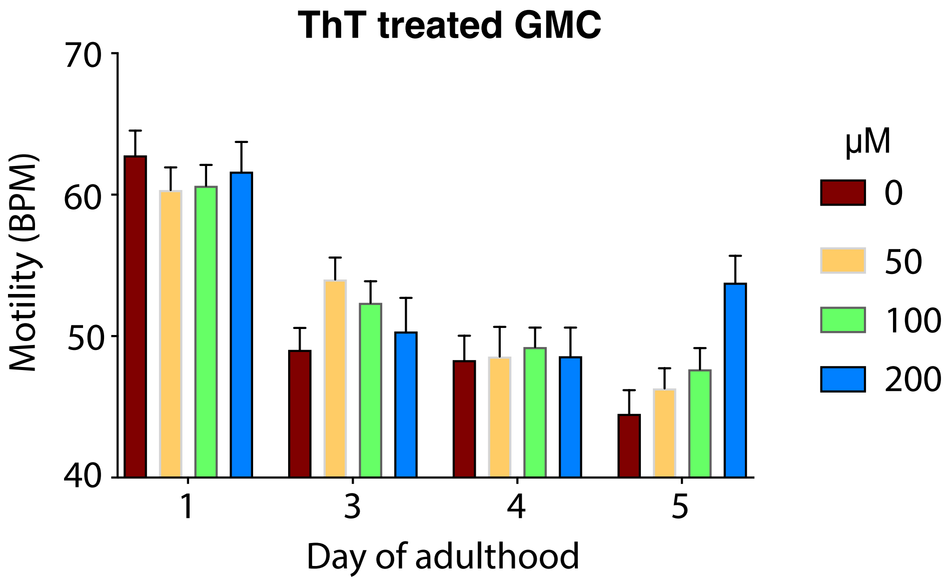

Figure S3. Kinetic profiles of the aggregation of a 2µM Aβ42 sample in the absence (black) and the presence of 20 µM, 50 µM, 100 µM and 500 µM concentrations of carnosine or kynurenic acid (represented in different colours). Note that this experiment was performed at the same time as shown in Fig. 4, and the kinetic profiles of Aβ42 only has been repeated on this plot for the purpose of clarity. Error bars are expressed as the standard deviations from 3 technical replicates.

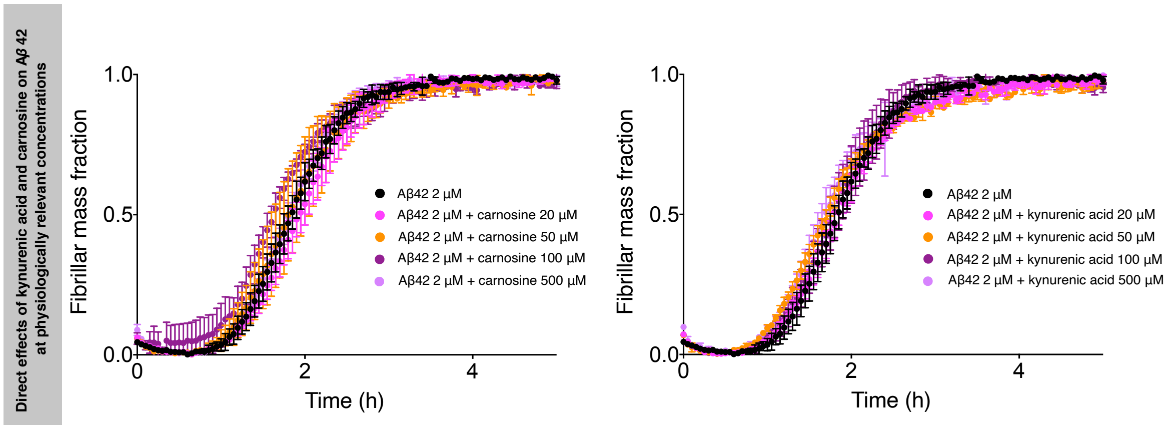

Figure S4. Carnosine and kynurenic acid do not show overt effects on the motility of normal wild type N2 worms. We tested a range of concentrations of carnosine (**a**) and kynurenic acid (**b**) on worm (N2) motility as a function of age (Days 3, 5 and 7), and did not observe any overt changes in motility. For clarity, total fitness score of Day 5 N2 worms is shown in (**c**).

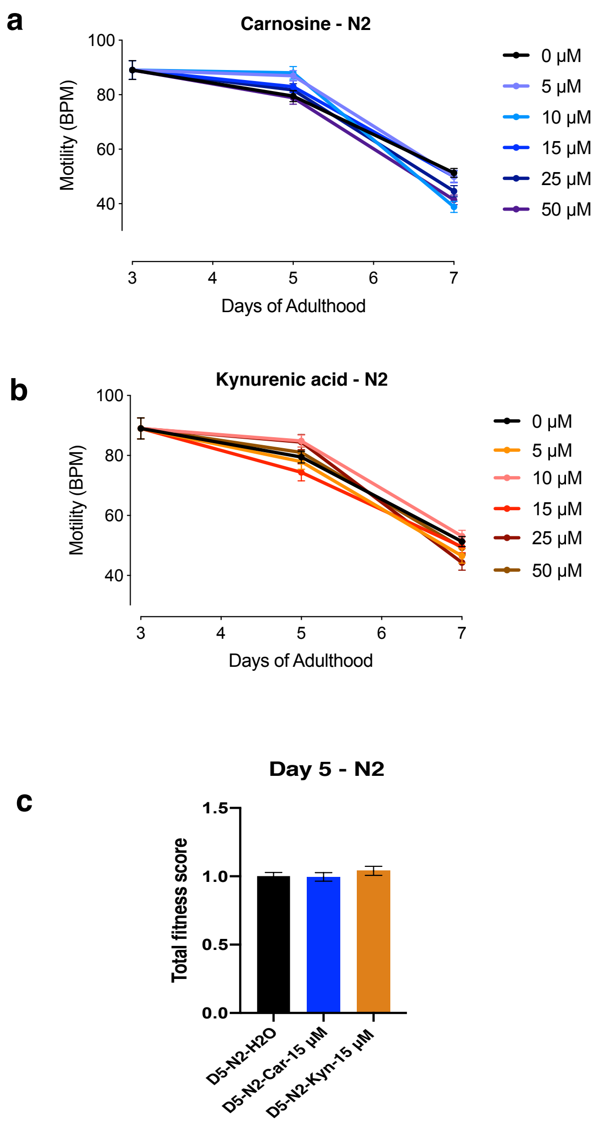

Figure S5. Carnosine and kynurenic acid activate a cytosolic unfolded protein response through a HSF-1 dependent mechanism. Uncropped full blots supporting data in Figure 5. (a) HSF-1 (b) HSP-90 (c) HSP-70 (d) DNJ-12 (e) DNJ-13 (f) DNJ-19.

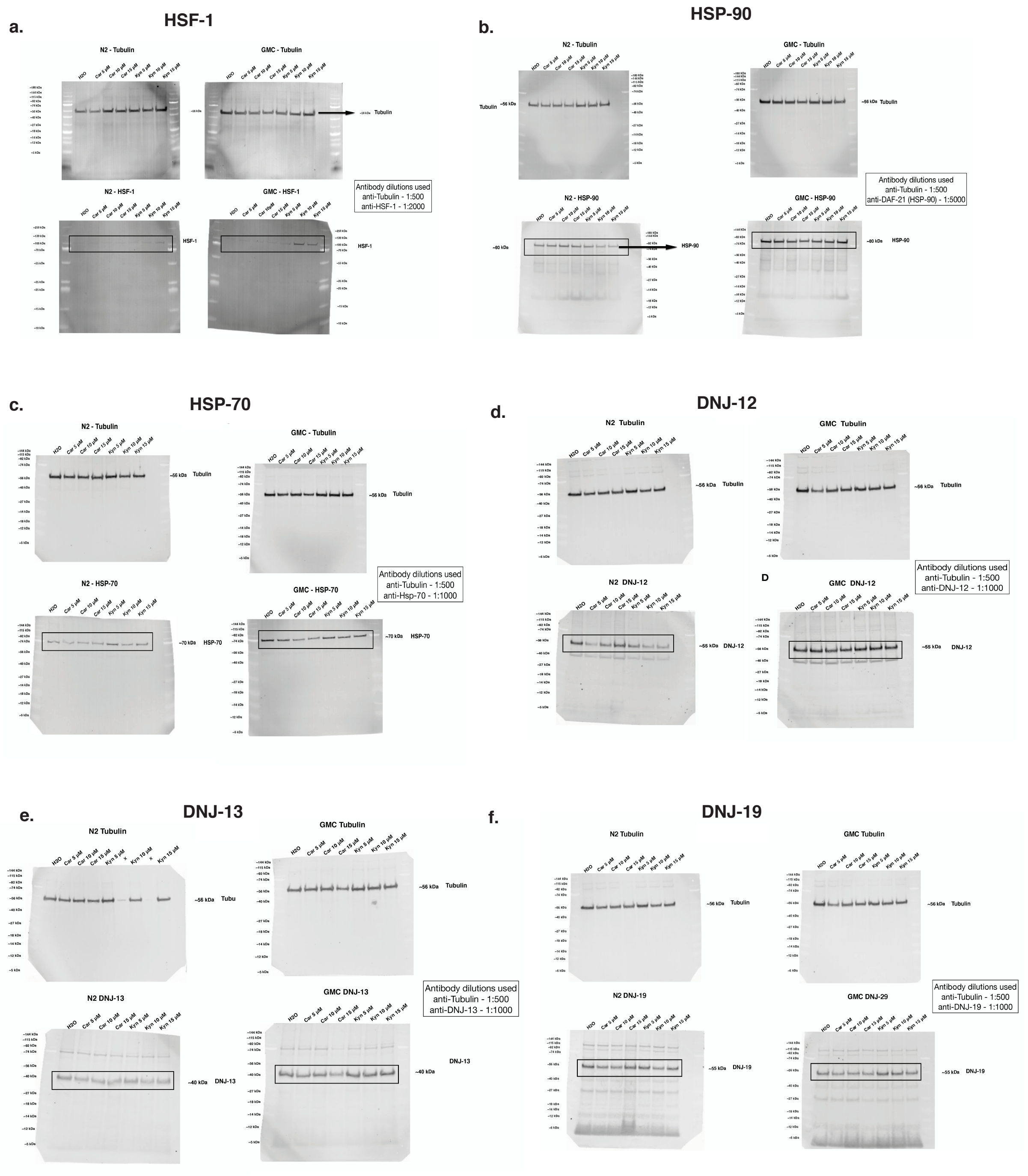

References

[1] Alavez, S., et al., Amyloid-binding compounds maintain protein homeostasis during ageing and extend lifespan. Nature, 2011. **472**(7342): p. 226-9.
