# Supplementary material for: Two human metabolites rescue a C. elegans model of Alzheimer’s disease via a cytosolic unfolded protein response
Source: Commun Biol. 2021 Jul 7;4:843. doi: 10.1038/s42003-021-02218-7 (PMC8263720; doi:10.1038/s42003-021-02218-7)
Supplement: Supplementary file 2 — Description of Additional Supplementary Files [file 42003_2021_2218_MOESM2_ESM.pdf]

**Description of Additional Supplementary Files**

**File name:** Supplementary data 1
**Description:** Dysregulated Metabolites in protein misfolding diseases.

**File name:** Supplementary data 2
**Description:** Source data for Figure 3-8.
